# Repetitive Transcranial Magnetic Stimulation for Action Naming in Aphasia Rehabilitation: A Systematic Review and Meta-Analysis

**DOI:** 10.3390/brainsci14070665

**Published:** 2024-06-29

**Authors:** Manon Spigarelli, Audrey Lalancette, Hugo Massé-Alarie, Maximiliano A. Wilson

**Affiliations:** 1 Centre Interdisciplinaire de Recherche en Réadaptation et Intégration Sociale—CIRRIS, 525 Bd Wilfrid-Hamel, Québec, QC G1M 2S8, Canada; audrey.lalancette.2@ulaval.ca (A.L.); hugo.masse-alarie@fmed.ulaval.ca (H.M.-A.); maximiliano.wilson@fmed.ulaval.ca (M.A.W.); 2 École des Sciences de la Réadaptation, Faculté de Médecine, Université Laval, 1050 Av. de la Médecine, Québec, QC G1V 0A6, Canada

**Keywords:** aphasia, rehabilitation, rTMS, action naming, speech therapy

## Abstract

Anomia, characterized by difficulty in word retrieval, particularly action verbs, poses a significant challenge in post-stroke aphasia. Repetitive transcranial magnetic stimulation (rTMS) has gained attention for language processing investigations and interventions. This systematic review explores the potential of rTMS as a modality to address action-verb deficits in post-stroke aphasia. We searched MEDLINE via PubMed, CINAHL via Ebsco and Web of Science in February 2024 for English articles (1996–2024). Eligible studies involved post-stroke aphasia action naming rehabilitation with rTMS. In some of these studies, rTMS was combined with speech-language therapy. In total, 10 studies were included in this systematic review. These articles highlight the potential of rTMS in improving verb retrieval deficits. While significant improvements may not be evident, notable progress both before and after intervention is observed in this review. However, it also underscores the need for further research to enhance language recovery for individuals with post-stroke aphasia.

## 1. Introduction

Anomia is defined as the difficulty in finding the right and appropriate word to name objects, people, and actions [1]. This deficit is one of the most significant cognitive complaints in individuals with post-stroke aphasia [2]. Indeed, anomia is nearly universal in aphasia [3]. It is considered one of the most frustrating and distressing aspects of the condition [4]. Anomia has been shown to be more pronounced for action verbs (e.g., run) than for objects (e.g., shoes). This can be explained from a linguistic perspective, notably due to the greater cognitive cost required to process action verbs compared with nouns [5,6,7]. Indeed, in a morphologically complex language such as French or Spanish, verbs are conjugated based on tense, mood, voice, person, number, and sometimes gender. Consequently, there are numerous inflected forms for a single verb [8]. In contrast, common nouns only change in gender and number. A verb expresses a state, an attitude, or an action. In the present review, we focus on action verbs. Action verbs are thus a subcategory of verbs that specifically depict an activity, movement or process performed by someone or something [9].

Verbs can be clinically distinguished from nouns, as demonstrated by various neuropsychological studies that support the hypothesis of a double dissociation between these word categories. For example, Goodglass et al. showed that patients with Broca’s aphasia achieved higher scores in naming objects compared with actions [10]. Similarly, Miceli et al. observed that individuals with agrammatism experienced more difficulties in verb naming, while persons with anomia had more difficulty naming nouns [11]. Numerous subsequent studies have confirmed this distinction between nouns and verbs [8,12,13]. Damasio and Tranel reported two patients with temporal lobe damage who had specific difficulties retrieving nouns and one patient with damage to the posterior segment of the inferior frontal gyrus who had difficulties retrieving verbs [14]. In another study, Aggujaro et al. examined lesion localization in 20 aphasic patients with disproportionate impairments in either nouns or verbs [15]. They found no verb-impaired patients with pure frontal damage; instead, several cases involved isolated left posterior-temporal and inferior-parietal brain damage. 

While neuropsychological evidence supports distinct neural pathways for nouns and verbs, functional neuroimaging studies have yet to clearly determine whether these neural structures are anatomically distinct in the brain [14]. Studies using functional magnetic resonance imaging (fMRI) have pinpointed the left prefrontal areas, the lateral temporal cortex (particularly the posterior middle temporal gyrus (MTG) and the inferior frontal cortex), the parietal lobe, the fusiform gyrus, and premotor cortex as regions displaying increased activation during verb processing compared with nouns [16,17,18,19,20]. This network is predominantly left-lateralized. The processing of verbs is suggested to engage the prefrontal and premotor cortices to a greater extent compared with noun processing [21]. These distinct neural networks for nouns and verbs have direct implications for the assessment and rehabilitation of patients with aphasia.

Anomia for action verbs is primarily assessed in speech therapy and neuropsychology using an oral action naming task, during which the individual is presented with an image depicting an action (e.g., a person running) and answers the question “What is the person doing?”. Action verbs play a critical role in the construction of sentences, influencing both the syntactic and semantic aspects of communication [22]. Recently, speech language therapy (SLT) for action verb anomia has gained significant attention due to the recognition of the importance of verbs in sentence comprehension and production (e.g., [22,23,24]). Improved verb retrieval can positively impact everyday language use (e.g., [25,26,27]). According to a literature review conducted by Webster and Whitworth [28], SLT targeting the recovery of verb naming ability in isolated contexts is as effective as speech-language therapy using sentence contexts. Consequently, enhancing the retrieval of verbs remains a primary goal of SLT. Diverse therapeutic approaches have been used to improve action naming [24]. These include the observation of actions [29], semantic–phonological strategies [30], naming to definition [31], and the semantic feature analysis (SFA) approach [27].

Novel approaches, such as transcranial magnetic stimulation (TMS), are now looked upon as they may contribute to improving anomia for action verbs [32,33]. For instance, Cotelli et al. [33] demonstrated that repetitive TMS (rTMS) improved action naming in individuals with the non-fluent variant of primary progressive aphasia following modulation of the dorsolateral prefrontal cortex. TMS is a neurostimulation and neuromodulation technique based on the principle of electromagnetic induction of an electric current in the brain through a brief magnetic field delivered via a stimulation coil held on the scalp over the targeted brain area [34]. While single TMS pulses are mainly used to investigate the function of motor areas by depolarizing the intracortical interneurons and corticospinal projections to muscles by recording the evoked motor responses [35], rTMS have been found to have long-lasting effects on the excitability of the targeted cortical areas [36,37]. For this reason, rTMS has been used for two main purposes: (i) to investigate the role of cortical areas by the modulation of their excitability (often named “virtual lesions”) to modify behaviors, and (ii) to improve the functioning of a cortical area which is altered due to a clinical condition (e.g., caused by a brain lesion) [38]. Thus, rTMS has been used to investigate language processing in healthy individuals and persons with brain damage [39,40]. A long-term beneficial effect of rTMS has been previously demonstrated in language processing among individuals with brain damage [41,42,43,44,45]. In line with this, the meta-analysis conducted by Arheix-Parras et al. highlights the promising potential of rTMS as a tool for the rehabilitation of individuals with post-stroke aphasia [39]. Furthermore, rTMS combined with SLT has shown superior performance compared with SLT alone in studies investigating the oral naming of images of objects [46]. Building on these advancements, this systematic review and meta-analysis aims to assess the efficacy of rTMS for the rehabilitation of action-verb anomia in post-stroke aphasia. Our review specifically focuses on experimental studies that use rTMS, either as a standalone intervention or in conjunction with SLT. Previous reviews have mostly covered a broad spectrum of language aspects, including oral production, fluency, and comprehension. However, none has specifically focused on action oral naming. A systematic review focusing on action verb anomia rather than anomia, in general, will provide a comprehensive portrait of the unique challenges individuals face in accessing and employing action verbs. This review aims to inform future protocols for post-stroke aphasia rehabilitation.

## 2. Materials and Methods

### 2.1. Study Selection and Analysis

We followed the four recommended steps for conducting systematic reviews: (1) formulating the review objective; (2) defining eligibility criteria; (3) conducting a search of the scientific literature; (4) selecting studies based on abstracts and then full texts [47]. This systematic review adheres to the PRISMA guidelines (http://www.prisma-statement.org). The detailed protocol was not recorded in a public register before data extraction began. 

We searched the MEDLINE database via PubMed, the CINAHL database accessed through Ebsco, and the Web of Science database in February 2024 for articles published between 1996 and 2024 in English and/or French. The following keywords and phrases were used to search titles, abstracts, and keywords: “aphasia” AND “repetitive transcranial magnetic stimulation” OR “rTMS” OR “non-invasive brain stimulation” (NIBS) OR “NIBS.” The search terminology and equations are presented in the Supplementary Materials.

The included studies met the following criteria:

1. Use of rTMS, including theta burst stimulation (TBS; i.e., rTMS using a specific frequency pattern).
2. Target population of individuals with post-stroke aphasia.
3. Articles written in French and/or English.
4. Treatment outcome measures included an oral action naming task that included action verbs.
5. The study aimed to improve the performance of patients with post-stroke aphasia.

Conversely, studies were excluded according to the following criteria:

1. Other forms of brain stimulation, such as transcranial direct current stimulation (tDCS) or other neuromodulation techniques, can be used.
2. Involvement of any other clinical pathology (e.g., primary progressive aphasia, Alzheimer’s disease, etc.) or healthy individuals.
3. Brain mapping during an action naming task.
4. The document was not a scientific journal article (e.g., commentaries, news articles, conference materials). Reviews and meta-analyses were not included, but their reference lists were consulted to identify articles not found in the initial search.

Two of the authors (AL and MS) independently examined the abstracts and titles of the articles identified by the database research to assess their suitability and inclusion in regard to the established selection criteria. Each study was assessed independently, and the full article was obtained for evaluation to determine if it met the inclusion/exclusion criteria. Any disagreements regarding the potential inclusion/exclusion of an individual document were resolved through discussion that reached a final consensus within the group of authors. Data extraction was conducted independently by two authors (AL and MS) using a standardized data collection form [48]. 

### 2.2. Assessment of the Methodological Quality of Included Studies

Two authors (AL and MS) independently assessed the methodological quality of each included article using the Quality Assessment Tool for Quantitative Studies developed by the Effective Public Health Practice Project (EPHPP; [49]), a critical appraisal tool including eight evaluation categories:

1. Selection bias
2. Study design
3. Confounders
4. Blinding procedures
5. Data collection methods
6. Withdrawals/dropouts
7. Intervention integrity
8. Analyses.

The quality of evidence for each evaluated category within the included articles is rated on a 3-point scale (1 point = strong quality, 2 points = moderate quality, and 3 points = weak quality). After analyzing the ratings of each evaluated component, an overall rating for the article is assigned. Any discrepancies were resolved through a final consensus among the evaluators.

### 2.3. Meta-Analyses

We collected pre- and post-rTMS intervention means, along with standard deviations and sample sizes, from action-naming tasks. In instances where numerical data were unavailable, the online tool WebPlotDigitizer was used to extract data from graphs when possible [50].

To determine the effect of rTMS on action verb anomia, studies comparing rTMS with a sham rTMS or with SLT were pooled using the RevMan 5.4 software. When the same outcome measures were used across studies, the mean difference (MD) was computed. Otherwise, a standardized mean difference was calculated to aggregate results using different types of outcome measures. Meta-analyses were computed at different time points (T2) after the end of the rTMS program (T1): short-term: 7 to 10 days post-rTMS; medium-term: 2 to 3 months post-rTMS; long-term: 8 months post-rTMS. We calculated standard deviations with RevMan when these were not explicitly provided in the articles. We expressed uncertainty using 95% confidence intervals (CI) and evaluated study heterogeneity via the I2 test at α = 0.05. A fixed-effect meta-analysis was conducted when the inconsistency index was I2 < 50%. Otherwise, a random-effect meta-analysis was employed.

If between-group meta-analyses were impossible to compute because of a lack of data, pre-post effects were calculated using RevMan 5.4 for each timepoint previously defined. A qualitative description of the results was employed to address the selected studies that could not be included in any of the meta-analyses.

## 3. Results

### 3.1. Study Selection 

**Figure 1:**
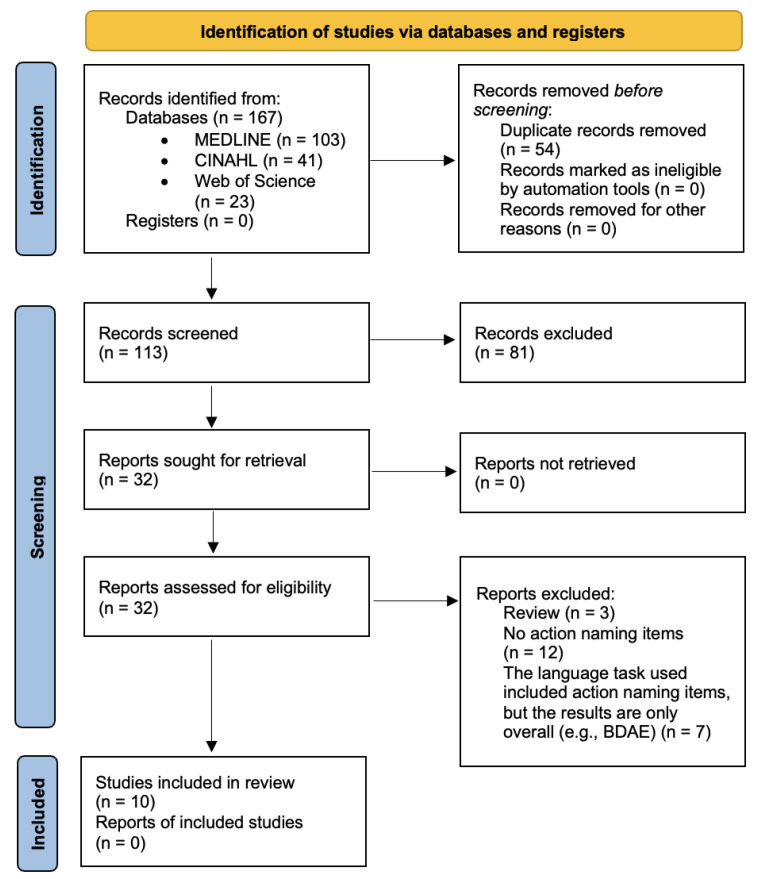
Flowchart of the Literature Search for the Meta-Analysis. From: [51].

A total of 167 relevant references were identified following the database search. After removing duplicates (*n* = 54), there were 113 articles left for evaluation. Based on the title and the abstract, 32 articles were selected for full-text review. After this step, 22 were excluded, resulting in a total of 10 included articles (Figure 1). All articles were written in English.

### 3.2. Description of the Included Articles

Among the ten included studies, only one focused specifically on action verbs. In the remaining nine studies, action verb naming was one of many outcome measures used. These studies predominantly emphasized other language tasks, such as oral object naming, oral description of images, and spontaneous discourse.

### 3.3. Quality Assessment

The assessment details for each study are presented in Table 1. The results indicate that most of the articles included in the systematic review show a weak methodological quality (rating of 3). The specific aspects that contributed to the low ratings are selection bias, study design, handling of confounders, blinding procedures, data collection methods, and the management of withdrawals and dropouts. Notably, some studies lacked information on intervention integrity and appropriate analysis aligned with the research question. Two articles, Heikkinen et al. [52] and Wang et al. [53], received higher ratings (rating of 1) than the eight others. These two studies had larger samples than the other studies in this analysis. 

### 3.4. Participants

Table 2 summarizes the characteristics of the participants across all studies in the rTMS condition. The mean age of participants across studies in the rTMS condition was 57.41 years, with an SD of 5.77. All of the participants had left middle cerebral artery (MCA) brain lesions. The mean time post-stroke of participants was 3.99, with an SD of 2.81. The studies were conducted in four different countries (Australia, the United States, Finland, and China) and had small sample sizes ranging from 1 [59,61] to 30 [53]. Overall, across the 10 studies reviewed, 68 patients with post-stroke aphasia received a real rTMS condition.

Six out of ten studies included in this review utilized a sham condition [52,53,54,55,57,58]. The participant groups in the rTMS and sham conditions within each study were similar in terms of age (mean age for sham condition: 62 years, SD: 3.41), time post-stroke (mean for sham condition: 2.95 years, SD: 1.42), and lesion site (left MCA territory) [52,53,54,55,57]. Note that in Garcia et al.‘s study [58], after the two-month visit, all patients initially in the sham condition were transferred to the real rTMS group. Overall, across the six studies reviewed, forty-one patients with post-stroke aphasia received a sham rTMS condition.

The remaining four studies [56,59,60,61] were single case studies. In this methodological design, each participant serves as their own control.

### 3.5. Brain Targets and Procedure 

The rTMS protocol employed across all 10 studies was similar. rTMS was administered at a frequency of 1 Hz, targeting the right IFG (Brodmann’s area (BA) 45 and BA44). The duration of each rTMS session was 20 min, with a total of ten sessions administered in nine studies (see Table 1 for details). Only one study conducted twenty sessions [52]. We did not find any articles that used another type of rTMS protocol, such as high-frequency rTMS or patterned rTMS (e.g., TBS). 

### 3.6. Outcome Measures 

Most studies (8 out of 10) used the Boston Diagnostic Aphasia Examination (BDAE; [62]) as their primary outcome measure. The BDAE includes a picture naming task comprising different word categories, such as objects, tools and implements, animals, and actions, with 12 images for each category. Action verbs were thus assessed with a relatively limited number of items. Heikkinen et al. employed the Action Naming Test (ANT; [63]) developed by Obler and Albert. The ANT contains 60 images depicting actions. Wang et al. employed 20 action pictures selected from the International Picture Naming Database [64].

### 3.7. Meta-Analyses 

Table 3 presents the outcome measure and the specific results obtained across time points for the included studies. Due to a lack of reported data within the available articles, it was not possible to calculate the efficacy of rTMS compared with a control group (e.g., sham rTMS or SLT) [52,53,57]. Given the limited availability of these data, we included in this meta-analysis only studies that provided both pre- and post-rTMS assessments without concomitant SLT [54,55,56,57,60]. Thus, only 5 out of the 10 studies were included in the meta-analysis [54,55,56,57,60]. The remaining five studies are presented in the qualitative analysis section. The study by Martin et al. [60] was included both in the meta-analysis and in the qualitative analysis section because of data availability for one participant but not the other.

#### 3.7.1. Short-Term rTMS Effects

**Figure 2:**
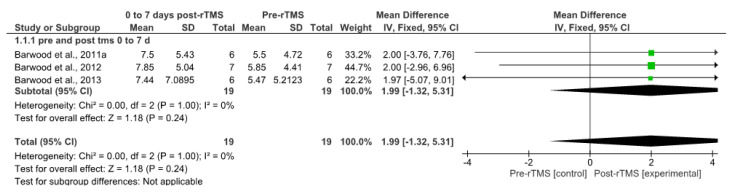
Forest Plot of Mean Difference and 95% CI For the Outcome of Action Naming in Patients Received rTMS at Short Term (0 to 7 Days) [54,56,57]. Note. Chi^2^, the value of the Chi-square test for heterogeneity; CI = confidence interval; IV = inverse variance; SD = standard deviation.

Results for short-term rTMS effects are summarized in Figure 2. The comparison between pre-test measures and measures taken 7 to 10 days after the rTMS intervention was assessed using data from three studies [54,56,57]. The meta-analysis showed a non-significant mean difference of 1.99 (95% CI: [−1.32, 5.31]; *p* = 0.24; I^2^ = 0%) in participants’ performance in pre- versus post-intervention. 

#### 3.7.2. Medium-Term rTMS Effects 

**Figure 3:**
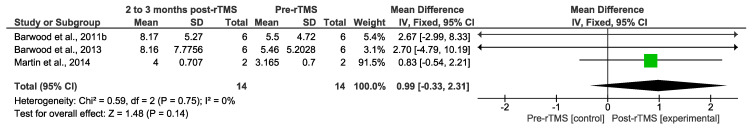
Forest Plot of Mean Difference And 95% CI for the Outcome of Action Naming in Patients Received rTMS at Middle Term (2 to 3 Months) [55,57,60]. Note. Chi^2^, the value of the Chi-square test for heterogeneity; CI = confidence interval; IV = inverse variance; SD = standard deviation.

The results for medium-term rTMS effects are summarized in Figure 3. The comparison between pre-test measures and measures taken 2 to 3 months after the rTMS intervention was assessed using data from three studies [55,57,60]. The meta-analysis indicated a non-significant mean difference of 1.48 (95% CI: [−0.33, 2.31]; *p* = 0.14; I^2^ = 0%). 

#### 3.7.3. Long-Term rTMS Effects

**Figure 4:**
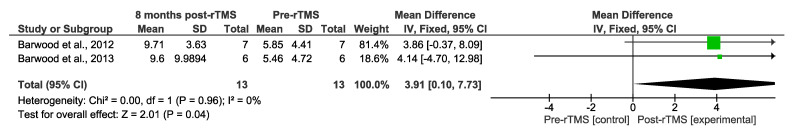
Forest Plot of Mean Difference and 95% CI for the Outcome of Action Naming in Patients Received rTMS at Long Term (8 Months) [56,57]. Note. Chi^2^, the value of Chi-square test for heterogeneity; CI = confidence interval; IV = inverse variance; SD = standard deviation.

The results for long-term rTMS effects are summarized in Figure 4. The comparison between pre-test measures and measures taken 6 to 8 months after the rTMS intervention was assessed using data from two studies [56,57]. The meta-analysis indicated a significant mean difference of 3.91 (95% CI: [0.10, 7.73]; *p* = 0.04; I^2^ = 0%). 

### 3.8. Qualitative Analysis

Here, we describe the studies that were not included in the meta-analysis. 

#### 3.8.1. Single Case Studies

Single case studies were excluded from the meta-analyses because of their sample size and lack of data, notably SD. Naeser et al.’s single-participant study showed better performance 6 months after the rTMS intervention (pre-rTMS mean: 3.33 (1.71); post-rTMS mean: 5) [61]. Hamilton et al.’s single case study exhibited a significant improvement between the pre-test (score obtained in pre-rTMS: 5) and 6 months post-rTMS measures (score obtained post-rTMS: 10) [59]. 

#### 3.8.2. Group Study

Garcia et al.’s study with nine participants showed decreased performance at 2 months post-rTMS (post-rTMS mean: 3.84), better performance at 6 months post-rTMS (post-rTMS mean: 10.8), as compared with pre-rTMS intervention (pre-rTMS mean: 4.92) [58]. This study could not be added to the meta-analysis due to a lack of data, notably SD.

#### 3.8.3. Speech-Language Therapy and rTMS Combination

Three studies investigated the effects of combining rTMS with some form of SLT. These studies were excluded from the quantitative analyses due to differences in SLT methods and insufficient data availability. Martin et al. conducted a study with two patients with post-stroke aphasia, one with severe non-fluent aphasia and the other with mild-to-moderate non-fluent aphasia [60]. Participants received rTMS sessions coupled with SLT. Precisely, modified Constraint-Induced Language Therapy (mCILT [65]), also known as intensive language-action therapy (ILAT), was used. Following each of the 20-min rTMS sessions, one hour of mCILT was immediately administered. After a one-hour lunch break, a further two hours of mCILT were administered. The daily total was 3 h of mCILT intervention. During each mCILT treatment session, an opaque screen was positioned on the table between the clinician and the patient, allowing only eye contact above the screen. A window in the screen facilitated the exchange of picture cards. The patient’s task was to name the picture on each card verbally. Gestures, writing, and sound effects were strictly prohibited during the naming process. Pictures were categorized by naming frequency, with higher frequency pictures introduced first and difficulty increasing gradually over 10 sessions. Therapy included sets of “always-named,” “sometimes-named,” and “never-named” pictures. Martin et al. found significant improvement in the patient with severe nonfluent aphasia for both measurement times (1 month and 6 months) (Score obtained pre-rTMS: 4.33 (0.58); score obtained at 1 month post-rTMS: 6 and at 6 months post-rTMS: 7), whereas the analysis did not reach significance in the patient with mild-to-moderate nonfluent aphasia (Score obtained pre-rTMS: 3.00 (0.58), the score obtained at 2 months post-rTMS: 3 and at 16 months post-rTMS: 3) [60]. The effects of the combination of rTMS and SLT were better than those of rTMS alone. 

Heikkinen et al. also coupled rTMS with SLT, the ILAT [66] in patients with post-stroke aphasia in the chronic stage (nine with real rTMS and eight with sham) [52]. Patients were administered the ILAT intervention daily for 3 h for a total of 30 h during 10 days (5 days per week for 2 weeks). ILAT involves communicative language games conducted in a small-group setting. Participants, consisting of three individuals with aphasia and one speech-language therapist, are seated around a table with their own sets of picture cards. Barriers are placed to prevent participants from seeing each other’s cards. Each participant has two copies of object or action picture cards in their set. The therapy session proceeds with participants taking turns making verbal requests. One player selects a card from their set and requests the corresponding card from another participant, promoting verbal interaction and communication skills practice. ILAT sessions were administered either directly following rTMS or sham stimulation. Action naming scores improved significantly after ILAT speech intervention in both groups (rTMS or sham). The addition of the rTMS condition had no significant effect [52].

In an RCT, Wang et al. tested the combination of rTMS with SLT in individuals with chronic nonfluent aphasia (*N* = 45) [53]. In their research, participants were divided into three groups: the real rTMS group with synchronous action naming, the real rTMS group with subsequent action naming, and the sham rTMS group with synchronous action naming. Each participant received a 60-min session with SLT twice a week and some naming training that was administered after the rTMS. The SLT session focused on improving verbal expressive skills through repetition, phonemic training, semantic training, naming exercises, conversation practice, picture-description tasks, and phrase-generation activities. Participants were instructed not to use compensatory methods such as gestures, drawing, or intonation of melodies during both the training sessions and self-learning activities. Their results suggest that the effect of rTMS coupled with simultaneous naming training was superior in improving action naming (naming accuracy: baseline: 36.6%; post-rTMS: 46.6%; *p* < 0.05) compared with subsequent naming training with rTMS (naming accuracy: baseline: 30.5%; post-rTMS: 33.8%), or synchronous naming training with placebo rTMS (naming accuracy: baseline: 34.6%; post-rTMS: 32%). 

The selected articles show heterogeneity in speech therapy approaches, which will need to be considered in interpreting the results.

## 4. Discussion

The aim of this literature review was to gather studies focusing on the effect of rTMS on action naming in individuals with post-stroke aphasia. Our objective was to analyze and synthesize available data to gain a better understanding of how this technique affects the ability to name actions. We sought to assess the consistency of the observed effects and the potential benefits of the rTMS stimulation regarding language processing. 

The results of the present meta-analysis show a trend towards better action naming performances after rTMS in individuals with chronic aphasia. Nevertheless, this improvement does not reach statistical significance for short (7 to 10 days post-rTMS) and middle terms (2 to 3 months post-rTMS). Conversely, rTMS significantly improved action naming in individuals with chronic aphasia in the long term (8 months post-rTMS). The qualitative analysis of the studies not included in the meta-analysis revealed similar results: significant long-term improvements (6 months post-rTMS), with no improvement in the middle term (2 months post-rTMS) [58,59]. Only Naeser et al.’s study showed no significant effect of rTMS in both middle-term (3 months post-rTMS) and long-term (6 months post-rTMS) periods [61]. 

The significant improvement observed at 6 months post-rTMS, without short-term effects, contradicts the findings reported in the current scientific literature. Indeed, significant effects are typically found immediately after treatment [44,67,68]. Some studies suggest the maintenance of language improvements up to 3 months post-rTMS, depending on the protocol used [69,70,71]. The few studies that found a maintenance of the rTMS effect after 3 months have previously found this effect in previous time points [45,72]. Bucur and Papagno’s meta-analysis [73] found no significant differences between data collected immediately after treatment and during follow-up (up to 6 months) for naming rehabilitation, suggesting sustained treatment effects over time with minimal decline or improvement.

Thus, we find these long-term effects puzzling and difficult to reconcile with the current literature. One possible explanation might come from the neuroimaging literature. Neuroimaging studies [74,75,76] employing positron emission tomography (PET) have demonstrated that 1 Hz rTMS, combined with SLT, induces long-term changes in brain activation patterns. These studies have shown a shift in activation towards preserved regions of the left hemisphere, correlating with improved language function in subacute post-stroke aphasic patients. This supports the idea that rTMS may facilitate the gradual reorganization and restoration of more effective neural networks over time, although these studies also indicate an immediate improvement following stimulation. The findings of this meta-analysis thus contrast with the current literature. In fact, rTMS is known to influence the secretion of brain-derived neurotrophic factor (BDNF), a protein critical for developing new neural pathways in the brain [77]. This neurotrophic effect may contribute to the observed long-term improvements by supporting neuroplasticity and neural function recovery after injury [78]. Techniques such as rTMS have the potential to induce lasting changes in the brain. However, the overall low methodological quality of the studies included in this meta-analysis limits the ability to draw definitive conclusions. Conducting and replicating longitudinal studies are essential to establish clear rehabilitation guidelines for addressing verb anomia in stroke patients. Additionally, caution is advised when interpreting the studies by Barwood et al. [54,55,56,57] as they appear to have significant overlap in patient characteristics across multiple studies, suggesting potential reliability issues.

In addition, several studies investigated the combination of rTMS with SLT. The studies in the present systematic review showed no significant effect of rTMS, as compared with sham, when combined with different SLT protocols in patients with aphasia [52,60]. In both cases where patients received this treatment combination, significant results were found due mainly to speech therapy interventions. Conversely, Wang et al. found that the treatment combination led to enhanced action naming compared with rTMS or SLT alone [53]. This finding is consistent with the existing literature on rTMS regarding object naming. For instance, a meta-analysis indicated that rTMS combined with SLT offered superior improvements in object naming scores compared with placebo rTMS combined with SLT or SLT alone [46]. These results highlight the need to conduct further studies with increased sample sizes that rely on randomized controlled trials to find out whether rTMS coupled with SLT produces a synergic effect for language recovery. 

Regarding stimulated cortical zones, all the studies presented here investigated the outcomes of rTMS stimulation of the right IFG on action naming homolog of Broca’s area. Ren et al.’s meta-analysis showed the efficacy of low-frequency rTMS applied to the right IFG pars triangularis in patients with aphasia [78]. This intervention notably amplified overall language function and different linguistic tasks, including naming, repetition, writing, and comprehension. A randomized, double-blind, placebo-controlled study examined changes in language performance after stimulation of Broca’s (i.e., right IFG) or Wernicke’s homologs with low-frequency rTMS in patients with aphasia [79]. Both the stimulation of Broca’s and Wernicke’s areas showed greater improvements in language scores compared with sham groups. The inhibition of the right IFG pars triangularis improved performance in spontaneous speech and repetition. This inhibition of the right IFG, therefore, looks promising for enhancing oral naming performance in patients with aphasia. On the other hand, the results of the current meta-analysis show that applying rTMS on the right IFG has no short-term effect on action naming in patients with post-stroke aphasia. This randomized study was conducted between 4 and 12 weeks post-stroke. Conversely, in the articles included in our systematic review and meta-analysis, the post-stroke onset average was around 3 years. The duration since stroke onset plays a crucial role in rehabilitation outcomes [80,81,82]. This difference in stroke onset may explain the different results found in our study and those of Ren et al. [79]. Future research should consider these temporal dynamics more rigorously, employing longitudinal designs to elucidate optimal treatment windows and refine therapeutic protocols for individuals with post-stroke aphasia.

Interestingly, no study has explored the stimulation of the left IFG on action naming. It consists of BA44 (pars opercularis of the IFG) and BA45 (pars triangularis of the IFG). The left IFG, possibly including some homologous areas in the right hemisphere, is known to be engaged in conceptual knowledge [83,84]. In certain types of aphasia (i.e., Wernicke’s), the left IFG may remain unaffected by lesions within the left hemisphere [85]. Therefore, it would be informative to investigate the effects of rTMS applied to the left IFG on action naming in such cases [85]. In addition, verb processing mainly involves the prefrontal and premotor cortices, compared with noun processing. While the dorsolateral prefrontal cortex (DLPFC) was not specifically investigated in any of the eligible studies, numerous studies have consistently supported the facilitatory effects of rTMS applied on this region on action naming in other neurological populations. For instance, Cotelli et al. demonstrated improved action naming performance during high-frequency rTMS applied to the left and right DLPFC in patients with probable Alzheimer’s disease [32]. Given the promising findings from related studies, further research focusing on the left IFG and the DLPFC in the context of post-stroke aphasia could yield valuable insights and is, thus, an avenue worth investigating.

### Limitations and Future Directions

There are several limitations that should be addressed within the context of this study. First and foremost, only five out of ten studies are randomized controlled trials. This poses a significant methodological challenge as non-controlled studies or case studies may harbor potential biases in how results are obtained and interpreted. The use of randomized controlled trials would allow for studies with stronger methodological qualities and more generalizable conclusions.

It is also imperative to elucidate the notion of sham or placebo. Sham or placebo in rTMS serves as a control, enabling researchers to account for the psychological and physiological effects of the placebo. Participants are generally unaware of whether they are receiving active or sham rTMS. This reduces the bias in reporting perceived effects. Half of the studies presented here did not use a placebo condition. This renders it difficult to discern the true effect of rTMS. 

Furthermore, only one out of the ten studies specifically focused on the effects of rTMS on action naming. It appears of paramount importance to use a specific action naming task rather than solely relying on a subtest from a language battery. All the studies presented here used images to elicit action naming. Action naming has been shown to improve when using videos depicting actions rather than images [86,87]. Unlike static images, videos offer a more comprehensive representation of actions, enabling individuals to access a richer network of semantic associations and motor-related knowledge, leading to enhanced action-naming abilities [88].

Additionally, several studies reviewed in this meta-analysis were conducted by the same authors and involved participants with strikingly similar characteristics [54,55,56,57]. These substantial similarities should be carefully considered, as they may limit the reliability and generalizability of the findings. Another limitation pertains to the relatively small number of participants included in each study. The restricted sample sizes could constrain the extent of generalizability and the representativeness of findings for the entire population. 

Finally, only a low-frequency rTMS (1 Hz) protocol for 20 min was used in the studies reported here. Dammekens et al. demonstrated that high-frequency rTMS (10 Hz) applied to the left IFG improved naming in patients with non-fluent aphasia [69]. The exploration of other rTMS protocols would enrich our understanding of action naming.

## 5. Conclusions

In summary, this systematic review and meta-analysis provides a comprehensive overview of the current literature regarding the effect of rTMS on action naming in patients with post-stroke aphasia. The results of the meta-analyses show that, despite a trend towards better action naming performances after rTMS, the changes in action naming were not statistically significant. The comparison of pre-post-rTMS was significant only for the 6-to-8-month post-rTMS period. Future studies should adopt more standardized methodologies, encompass larger and more diverse samples, employ more appropriate outcome measures, and consider comparative analyses across different populations.

## Figures and Tables

**Table 1:** Summary of Studies Investigating the Effects of rTMS on Action Naming in Patients with Chronic Post-Stroke Aphasia.

Study	Design	Participants	Stimulation Procedure	Sessions	Control Condition	Speech Training	Outcome Measures	Critical Appraisal						
								Selection Bias	Study Design	Confounders	Blinding	Data Collection Methods	Withdrawals and Drop-Outs	Global Evaluation
Barwood et al., 2011A [54]	Randomized controlled trial	12	rTMS at 1 Hz on R IFG (pars triangularis) for 20 min	10	Sham coil	N/A	BDAE	1	1	3	1	1	3	3
Barwood et al., 2011B [55]	Randomized controlled trial	12	rTMS at 1 Hz on R IFG (pars triangularis) for 20 min	10	Sham coil	N/A	BDAE	1	1	3	1	1	3	3
Barwood et al., 2012 [56]	Cohort study	7	rTMS at 1 Hz on R IFG (pars triangularis) for 20 min	10	N/A	N/A	BDAE	2	1	3	3	1	3	3
Barwood et al., 2013 [57]	Randomized controlled trial	12	rTMS at 1 Hz on R IFG (pars triangularis) for 20 min	10	Sham coil	N/A	BDAE	2	1	1	1	1	3	2
Garcia et al., 2013 [58]	Non-randomized study	9	rTMS at 1 Hz on R IFG (pars triangularis) for 20 min	10	Sham coil	N/A	BDAE	3	1	3	2	1	3	3
Hamilton et al., 2010 [59]	Case-control study	1	rTMS at 1 Hz on R IFG (pars triangularis) for 20 min	10	N/A	N/A	BDAE	3	2	N/A	3	1	N/A	3
Heikkinen et al., 2019 [52]	Randomized controlled trial	17	rTMS at 1 Hz on R IFG (pars triangularis) for 20 min	20	Sham coil	Intensive Language-Action Therapy: 3 h of intensive (total of 30 h)	ANT	2	1	1	2	1	1	1
Martin et al., 2014 [60]	Case study	2	rTMS at 1 Hz on R IFG (pars triangularis) for 20 min	10	N/A	Massed Constraint-Induced Language Therapy (mCILT): 3 h of intensive	BDAE	3	2	3	3	1	N/A	3
Naeser et al., 2010 [61]	Case study	1	rTMS at 1 Hz on R IFG (pars triangularis) for 20 min	10	N/A	N/A	BDAE	3	2	N/A	3	1	3	3
Wang et al., 2014 [53]	Randomized controlled trial	45	rTMS at 1 Hz on R IFG (pars triangularis) for 20 min	10	Sham coil	Speech training twice a week + online or offline naming training	International Picture Naming Database	1	1	1	1	1	1	1

Note. R = right; IFG = inferior frontal gyrus; min = minutes. Boston Diagnostic Aphasia Examination (BDAE; [62]). Action Naming Test (ANT; [63]). International Picture Naming Database [64].

**Table 2:** Participant Characteristics Across Selected Studies for rTMS group.

Study	Mean Age (Years) (SD)	Age Range (Years)	TPO (Years) (SD)	Educational Level (Years) (SD)	Men:Women	Country	Lesion Location	Aphasia Type
Barwood et al., 2011A [54]	60.83 (5.98)	54–67	3.7 (1.26)	13.3 (3.2)	4:2	Australia	Left MCA	Moderate to severe non-fluent
Barwood et al., 2011B [55]	60.83 (5.98)	54–67	3.7 (1.26)	13.3 (3.2)	4:2	Australia	Left MCA	Moderate to severe non-fluent
Barwood et al., 2012 [56]	59.85 (6.04)	54–67	3.5 (1.25)	13 (3.11)	5:2	Australia	Left MCA	Moderate to severe non-fluent
Barwood et al., 2013 [57]	60.83 (5.98)	54–67	3.7 (1.26)	13.3 (3.2)	4:2	Australia	Left MCA	Moderate to severe non-fluent
Garcia et al., 2013 [58]	N/A	18–75	≥0.5	N/A	N/A	United States	Left hemisphere (spares SMA)	Mild to moderate non-fluent
Hamilton et al., 2010 [59]	61	61	7	18	1:0	United States	Left MCA	Global aphasia: Mild non-fluent aphasia
Heikkinen et al., 2019 [52]	53.67 (9.83)	37–72	2.89 (1.33)	12	7:2	Finland	All lesions were in the left hemisphere (temporoparietal, frontotemporoparietal, frontoparietal, basal ganglia, periventricular white matter, insula and internal capsule)	7 ischemic and 2 hemorrhagic cases 3 anomic, 3 conduction, 2 Broca, 1 transcortical motor
Martin et al., 2014 [60]	55 (11.31)	47–63	8.5 (5.37)	N/A	1:1	United States	Left MCA (P1), left intracerebral hemorrhage (P2)	Mild-moderate non-fluent (P1) and severe non-fluent (P2)
Naeser et al., 2010 [61]	43	43	1.5	N/A	1:0	United States	Left MCA	N/A
Wang et al., 2014 [53]	61.7 (0.63)	N/A	1.35 (0.06)	11.85 (0.49)	27:3	China	Left MCA	16 Broca’s aphasia, 11 transcortical motor’s aphasia, 3 mild-global’s aphasia

Note. MCA: Middle cerebral artery.

**Table 3:** Action Naming Scores at Different Time Points Post-Stroke and Post-rTMS.

Study	Outcome Measure	Sample	Time Point	Results
Barwood et al., 2011A [54] (n = 12, 6 with real rTMS and 6 with sham)	BDAE—action naming task (/12)	Mean score (SD)	Pre-test rTMS	5.50 (4.72)
			1 week post rTMS	7.5 * (5.43)
			Pre-test Sham	6.66 (5.27)
			1 week post Sham	6.17 (4.87)
Barwood et al., 2011B [55] (n = 12, 6 with real rTMS and 6 with sham)	BDAE—action naming task (/12)	Participant 1—rTMS	Pre-test rTMS	10
			2 months post rTMS	12
		Participant 2—Sham	Pre-test Sham	11
			2 months post Sham	11
		Participant 3—rTMS	Pre-test rTMS	11
			2 months post rTMS	11
		Participant 4—rTMS	Pre-test rTMS	6
			2 months post rTMS	12
		Participant 5—Sham	Pre-test Sham	11
			2 months post Sham	11
		Participant 8—Sham	Pre-test Sham	8
			2 months post Sham	7
		Participant 9—rTMS	Pre-test rTMS	0
			2 months post rTMS	3
		Participant 10—rTMS	Pre-test rTMS	0
			2 months post rTMS	0
		Participant 11—Sham	Pre-test Sham	10
			2 months post Sham	7
		Participant 13—rTMS	Pre-test rTMS	6
			2 months post rTMS	11
		Participant 14—Sham	Pre-test Sham	0
			2 months post Sham	0
		Participant 15—Sham	Pre-test Sham	0
			2 months post Sham	0
	BDAE—action naming task (/12)	Mean score (SD) [calculated from raw scores]	Pre-test rTMS	5.50 (4.72)
			2 months post rTMS	8.17 (5.27) **
			Pre-test Sham	6.66 (5.27)
			2 months post Sham	6 (4.49)
Barwood et al., 2012 [56] (n = 7)	BDAE—action naming task (/12) [data from figures]	Participant 1	Baseline	10
			1 week post rTMS	11
			2 months post rTMS	12
			8 months post rTMS	12
		Participant 2	Baseline	11
			1 week post rTMS	11
			2 months post rTMS	11
			8 months post rTMS	11
		Participant 3	Baseline	6
			1 week post rTMS	11
			2 months post rTMS	12
			8 months post rTMS	12
		Participant 4	Baseline	8
			1 week post rTMS	10
			2 months post rTMS	11
			8 months post rTMS	11
		Participant 5	Baseline	0
			1 week post rTMS	1
			2 months post rTMS	3
			8 months post rTMS	8
		Participant 6	Baseline	0
			1 week post rTMS	0
			2 months post rTMS	0
			8 months post rTMS	2
		Participant 7	Baseline	6
			1 week post rTMS	11
			2 months post rTMS	11
			8 months post rTMS	12
	BDAE—action naming task (/12)	Mean score (SD) [calculated from raw scores]	Pre-test TMS	5.85 (4.41)
			1 week post rTMS	7.85 (5.04)
			2 months post rTMS	8.57 (4.92)
			8 months post rTMS	9.71 (3.63)
Barwood et al., 2013 [57] (n = 12)		Mean score	Baseline	[1,2,3] 5.47
			1 week post rTMS	7.44
			2 months post rTMS	8.16
			8 months post rTMS	9.62
			12 months post rTMS	9.95
Garcia et al., 2013 [58] (n = 9)	BDAE—action naming task (proportion correct)	Mean score	Baseline	0.41
			2 months post Sham	0.24
			2 months post rTMS	0.32
			6 months post rTMS	0.9
Hamilton et al., 2010 [59] (n = 1)	BDAE—action naming task (/12)	Participant 1	Baseline	5
			2 months post rTMS	6
			6 months post rTMS	10 *
			Chi-square test	(χ^2^ = 4.444; *p* = 0.035)
Heikkinen et al., 2019 [52] (n = 17, 9 with real rTMS and 8 with sham)	Action Naming Test (/60)	Mean score	Baseline rTMS	26 (4–49) (SD: 13.12)
			Baseline sham	52 (5–54) (SD: 18.30)
			*p*-values between groups	0.262
			Main time effect across groups	F(1,15) = 10.436; *p* = 0.001, n^2^ = 0.410
Martin et al., 2014 [60] (n = 2)	BDAE—action naming task (/12)	Participant 1	Baseline pre-rTMS mean	3.33 (1.71)
			3 months post-rTMS alone	4
			6 months post-rTMS alone	5
			Baseline pre-rTMS + mCILT	3.00 (0.58)
			2 months post-rTMS + mCILT	3
			16 months post-rTMS + mCILT	3
		Participant 2	Baseline pre-rTMS mean	3
			2 months post-rTMS alone	4
			4 years 3 months post-rTMS	3
			Baseline pre-rTMS + mCILT	4.33 (0.58)
			1 month post-rTMS + mCILT	6 ***
			6 months post rTMS + mCILT	7 ***
Naeser et al., 2010 [61] (n = 1)	BDAE—action naming task (/12)	Participant 1	Baseline 1 (14 months post-stroke)	5
			Baseline 2 (17 months post-stroke)	4
			Baseline 3 (17.5 months post-stroke)	1
			Pre-rTMS Entry Baseline mean (SD)	3.33 (1.71)
			3 months post-rTMS	4
			6 months post-rTMS	5
			29 months post-rTMS	2
			29 months post-rTMS	4
			29 months post-rTMS	3
Wang et al., 2014 [53] (n = 45)	20 action pictures selected from the Picture Naming Database (% accuracy ± SD)	rTMS + synchronous naming training (rTMSsyn)	Baseline	36.6 ± 18
			After rTMS treatment	46.6 ± 18.4 *^†^
			3 months post-treatment	53.8 ± 14.5 *^†^
		rTMS + subsequent naming training (rTMSsub)	Baseline	30.5 ± 18.2
			After rTMS treatment	33.8 ± 12.3
			3 months post-treatment	35.5 ± 14.7
		sham rTMS + synchronous naming training (rTMSsham)	Baseline	34.6 ± 16.1
			After rTMS treatment	32 ± 12.2
			3 months post-treatment	36.5 ± 12.0

Notes. rTMS—repetitive transcranial magnetic stimulation; SD—standard deviation. * *p* < 0.05, ** *p* < 0.01, *** Score increased at least 2 SD from baseline. ^†^ Significant difference between rTMSsyn and rTMSsub and between rTMSsyn and rTMSsham.

## Data Availability

The data presented in this study are available on request from the corresponding author due to privacy concerns regarding participant information (specify the reason for the restriction).

## Supplementary Materials

The following supporting information can be downloaded at: https://www.mdpi.com/article/10.3390/brainsci14070665/s1. Terminology and Search Terms.
