# Supplementary material for: Repetitive Transcranial Magnetic Stimulation for Action Naming in Aphasia Rehabilitation: A Systematic Review and Meta-Analysis
Source: Brain Sci. 2024 Jun 29;14(7):665. doi: 10.3390/brainsci14070665 (PMC11275163; doi:10.3390/brainsci14070665)
Supplement: Supplementary file 1 [file brainsci-14-00665-s001.zip › brainsci-3073967-supplementary.pdf]

Supplementary File

Medline via Ebsco
(MH aphasia) OR (TI aphasia) OR (AB aphasia)
AND
(MH "transcranial magnetic stimulation+") OR (TI "transcranial magnetic stimulation" OR "rTMS" OR "Non invasive brain stimulation" OR "NIBS" OR "TMS") OR (AB "transcranial magnetic stimulation" OR "rTMS" OR "Non invasive brain stimulation" OR "NIBS" OR "TMS")
AND
(MH "language tests+") OR (TI "naming") OR (AB "naming")

CINAHL via Ebsco
(MH aphasia) OR (TI aphasia) OR (AB aphasia)
AND
(MH "transcranial magnetic stimulation") OR (TI "transcranial magnetic stimulation" OR "rTMS" OR "Non invasive brain stimulation" OR "NIBS" OR "TMS") OR (AB "transcranial magnetic stimulation" OR "rTMS" OR "Non invasive brain stimulation" OR "NIBS" OR "TMS")
AND
(MH "language tests") OR (TI "naming") OR (AB "naming")

Web of Science
"Aphasia"
"transcranial magnetic stimulation" OR "rTMS" OR "Non invasive brain stimulation" OR "NIBS" OR "TMS"
"naming"
